# Transdermal Delivery of High Molecular Weight Antibiotics to Deep Tissue Infections via Droplette Micromist Technology Device (DMTD)

**DOI:** 10.3390/pharmaceutics14050976

**Published:** 2022-04-30

**Authors:** Lakshmi Pulakat, Howard H. Chen, Madhavi P. Gavini, Lauren A. Ling, Yinian Tang, Alexander Mehm, Gregory L. Martin, Corinna N. Beale, Brian P. Mooney, Hongmin Sun

**Affiliations:** 1 Tufts Medical Center, Molecular Cardiology Research Institute, Boston, MA 02111, USA; hchen1@tuftsmedicalcenter.org (H.H.C.); lauren.ling@tufts.edu (L.A.L.); ytang7@tuftsmedicalcenter.org (Y.T.); amehm@tuftsmedicalcenter.org (A.M.); gmartin@tuftsmedicalcenter.org (G.L.M.); 2 School of Medicine, Tufts University, Boston, MA 02111, USA; corinna.beale@tufts.edu; 3 Division of Cardiovascular Medicine, Department of Medicine, University of Missouri, Columbia, MO 65211, USA; 4 Droplette Inc., Boston, MA 02108, USA; madhavi@droplette.io; 5 Charles W. Gehrke Proteomics Center, Division of Biochemistry, University of Missouri, Columbia, MO 65211, USA; mooneyb@missouri.edu

**Keywords:** transdermal delivery, Droplette micromist technology device (DMTD), multidrug resistant bacteria, colistin methanesulfonate, vancomycin, *E. coli*, green fluorescent protein, live tissue imaging

## Abstract

Wound infection by multidrug-resistant (MDR) bacteria is a major disease burden. Systemic administration of broad-spectrum antibiotics colistin methanesulfonate (CMS) and vancomycin are the last lines of defense against deep wound infections by MDR bacteria. However, systemic administration of CMS and vancomycin are linked to life-threatening vital organ damage. Currently there are no effective topical application strategies to deliver these high molecular weight antibiotics across the stratum corneum. To overcome this difficulty, we tested if high molecular weight antibiotics delivered by Droplette micromist technology device (DMTD), a transdermal delivery device that generates a micromist capable of packaging large molecules, could attenuate deep skin tissue infections. Using green fluorescent protein-tagged *E. coli* and live tissue imaging, we show that (1) the extent of attenuation of deep-skin *E. coli* infection was similar when treated with topical DMTD- or systemic IP (intraperitoneal)-delivered CMS; (2) DMTD-delivered micromist did not spread the infection deeper; (3) topical DMTD delivery and IP delivery resulted in similar levels of vancomycin in the skin after a 2 h washout period; and (4) IP-delivered vancomycin was about 1000-fold higher in kidney and plasma than DMTD-delivered vancomycin indicating systemic toxicity. Thus, topical DMTD delivery of these antibiotics is a safe treatment for the difficult-to-treat deep skin tissue infections by MDR bacteria.

## 1. Introduction

The Infectious Diseases Society of America (IDSA) recognizes antimicrobial resistance as “one of the greatest threats to human health worldwide” [1]. In the US, annual additional costs linked to infections caused by drug resistant organisms as compared to susceptible organisms are estimated between USD 21 billion and USD 34 billion [1,2]. Skin and soft tissue infections (SSTIs) by multidrug resistant (MDR) organisms are major disease burden and affect people of all ages [3,4,5,6].

Complicated SSTIs caused by multidrug-resistant bacteria require treatment with strong antibiotics or their combinations. The guidelines of Infectious Disease Society of America suggest systemic treatment (oral/intravenous delivery) with broad-spectrum antibiotics such as colistin and vancomycin for complicated SSTIs [7]. Colistin is considered as the “last line of defense” drug against multidrug-resistant Gram-negative bacteria [8,9,10]. Often colistin or tigecycline are the only available treatments for MDR *A. baumannii* infections [11]. Systemic delivery of colistin and vancomycin is the standard-of-care to treat deep skin and chronic open wound infections by MDR bacteria because the efficacy of topical treatment is limited by the inability of antibiotics with sizes above ~500 daltons to penetrate the stratum corneum effectively (vancomycin is 1448 daltons and colistin is 1155 daltons) [12]. Moreover, most topically applied drugs are sweated or wiped off.

Unfortunately, systemic delivery of broad-spectrum antibiotics is often associated with life-threatening negative side effects including vital organ damage such as nephrotoxicity and hepatotoxicity [13,14,15,16,17]. These negative side effects further complicate patient recovery and increase the morbidity and mortality. The practice guidelines for vancomycin treatment for *Staphylococcus aureus* infection in adult patients recommend serum Vancomycin concentrations to be monitored to reduce the risk of nephrotoxicity in patients receiving aggressive treatment and patients with unstable renal functions [18]. It was found that the plasma colistin concentrations needed for antimicrobial activity could also increase the risk for nephrotoxicity [19,20]. Localized delivery of high molecular weight and broad-spectrum antibiotics deep into the skin is a much safer treatment approach that will avoid negative side effects. However, currently there are no effective transdermal delivery devices that are capable of delivering high molecular weight antibiotics such as colistin or vancomycin locally to deep skin infections and penetrate several millimeters deep into the skin to kill bacteria located in deep skin tissue.

**Figure 1:**
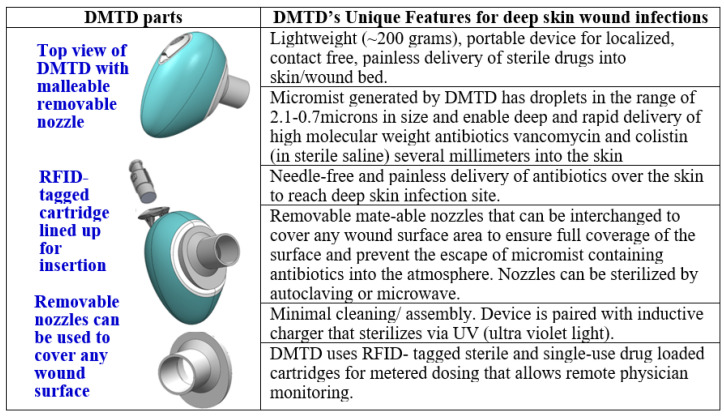
Unique characteristics of DMTD that allows localized transdermal delivery of high molecular weight antibiotics to treat deep skin infections and avoid systemic adverse side effects.

Droplette micromist technology [21] developed and patented (US 9,700,686 B2 and PCT/US2016/035695) by Droplette Inc. is a needle-free and painless transdermal delivery device. Droplette is the first device to combine a piezoelectric transducer and pneumatic diaphragm pump to provide delivery of large molecules deep into skin and soft tissue. The transducer cavitates the fluid to form uniform and monodisperse droplets ejected in a transitional-turbulent flow regime. The diaphragm pump then introduces force at an angle perpendicular to the flow, which reduces shear force on the droplets and accelerates their velocity, preventing coalescence of the droplets and inducing their break into even smaller sub-micron droplets that are capable of penetrating skin with high speed. We have been working with Droplette Inc. to develop a custom device called Droplette micromist technology device (DMTD) to treat deep skin infections. DMTD is fitted with a series of removable and mate-able nozzles of varying widths that can be interchanged to fully cover the wound surface area (open or closed wounds). These adjustable nozzles allow us to cover the surface area of the wound to allow rapid focused delivery of the micromist carrying antibiotics to the top of skin infection site to reach several millimeters deep into the skin. The unique characteristics of DMTD that makes it highly suited for treatment of wounds and deep skin infections are listed in Figure 1.

To determine whether DMTD-delivered micromist containing high molecular weight antibiotics such as colistin will treat deep skin infections as effectively as systemically delivered antibiotics, we used a rat model for deep skin infection. *E. coli* tagged with green fluorescent protein (GFP-*E. coli*) was used to visualize the depth of infection and the effects of treatment with colistin either via DMTD delivery on top of the skin infection site or via systemic delivery by intraperitoneal (IP) injection. To determine if DMTD-delivered micromist causes deeper penetration of bacteria, we also performed an experiment in which shallow wounds on rats were infected with GFP-*E. coli* and the depth of penetration of GFP-*E. coli* was assessed by live tissue imaging after DMTD delivery of saline. To compare tissue partitioning of high molecular weight antibiotics delivered via DMTD or IP, we used a mouse model to determine the levels of vancomycin delivered by DMTD or IP in the skin, kidney and serum after a 2 h washout period. Data presented here show for the first time that needle-free DMTD delivery of high molecular weight antibiotics can attenuate deep skin infections as effectively as IP delivery of the same antibiotic. Moreover, we show that while DMTD-delivered micromist reaches several millimeters deep into the skin, it does not cause deeper penetration of bacteria and skin concentration of antibiotic is similar when delivered by DMTD or IP.

## 2. Materials and Methods

### 2.1. GFP-E. coli Culturing

*E. coli* expressing green fluorescent protein (GFP-*E. coli*: 25922GFP) was purchased from American Type Culture Collection (ATCC) and single colonies were selected by plating on LB-agar (ThermoFisher Scientific, Waltham, MA, USA) containing ampicillin (100 µg/mL) (Sigma-Aldrich, St. Louis, MO, USA). Single colonies were sub-cultured in LB Broth with ampicillin and authenticated by determining GFP fluorescence using the IVIS Spectrum CT Biophotonic Imager (Perkin Elmer, Waltham, MA, USA). Liquid culture was concentrated by centrifugation and 2.3 × 10^9^ cells in 30 µL volume was used for deep skin infection.

### 2.2. Rat Wounding and Deep Skin Infection

Rats are a common model of wound healing due to their docile nature and small size for convenience of housing in animal experiments. Rat skin, similar to humans, has an epidermis, basement membrane and dermis layer [22]. All vertebrate animals were handled in accordance with US National Institutes of Health standards. All animal procedures (protocol B2020-56, approved 23 June 2020) were reviewed and approved by the Tufts Medical Center/Tufts University Institutional Animal Care Committee prior to commencement, and studies were thereafter closely monitored. Outbred female 5-month-old Wistar rats (from Charles River Laboratories, Wilmington, MA, USA) were maintained in the Tufts University animal facility in a reversed (12:12 h)-hour dark–light cycle so that the rat’s dark cycle (active time) is during the day. Rats were co-housed in individually ventilated cages (Thoren rat cage Model #8) and maintained on standard rat chow (Teklad©, 2918 Global 18% Protein Rodent Diet, irradiated bag) and acidified water ad libitum.

**Figure 2:**
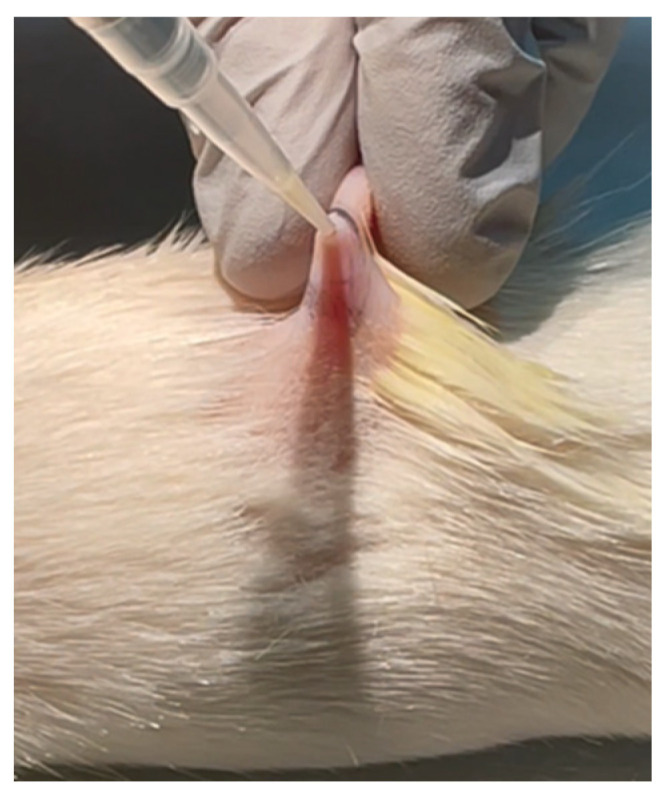
Representative image of generating deep skin infection on rat skin by dispelling 30 µL of saline containing 2.3 × 10^9^ GFP-*E. coli* cells into a 5 mm-deep wound generated by an 18-gauge needle.

Briefly, rats were anesthetized using isoflurane, and a 1 cm^2^ area of skin on the back was shaved and prepped with alcohol. Three deep but narrow wounds (5 mm deep) were generated on each rat’s back using an 18-gauge needle. Next, 30 µL of GFP-*E. coli* (2.3 × 10^9^ cells) was slowly dispelled into the narrow wound by pulling the skin upward and using a micropipette (Figure 2). Care was taken to ensure that all of the inoculum was dispelled into the wound.

### 2.3. Colistin Methanesulfonate (CMS) Treatment

**Figure 3:**
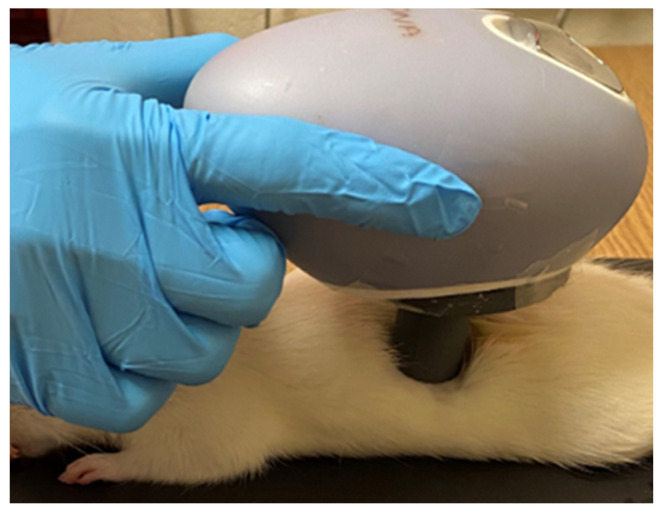
Representative image showing the placement of the nozzle of DMTD on top of the skin surrounding the infection site for delivery of CMS onto the third infected wound on a rat’s back.

Rats were anesthetized with isoflurane and colistin methanesulfonate (Cayman Chemicals) was used at a dose of 2.5 mg/kg in a volume of 300 µL of sterile saline for either IP injection or DMTD delivery. An equal amount of CMS in a 100 µL volume of sterile saline was used for topical application using a micropipette and the fluid was retained on top of the wound by generating a barrier around the wound using ophthalmic ointment (ndc24208-780-55). Sterile saline without CMS (300 µL) was used as vehicle control for IP injection and DMTD delivery. Rats receiving IP injection of saline (*n* = 3) served as controls for untreated wounds. Rats receiving IP injection of colistin methanesulfonate (*n* = 4) served as positive controls for drug treatment. DMTD delivery (Figure 3) was performed by placing the nozzle gently on the skin surrounding the top of the wound and thus allowing the micromist carrying CMS to penetrate into the wound and prevent escape into the atmosphere. DMTD delivery time for 300 µL was 30 s. In each rat, the first wound was treated with topical delivery of 100 µL of sterile saline containing CMS (retained on the top of the wound by the barrier to prevent loss); the second wound was treated with DMTD-delivered saline (vehicle control for DMTD delivery); and the third wound was treated with DMTD delivery of CMS (Figure 3). Topical delivery of CMS by the micropipette (referred as topical delivery hereafter) and DMTD delivery of CMS were carried out two hours after the wounding to allow time for the wounds to close partially. CMS and vehicle treatments were repeated once more 4 h after the initial treatment.

### 2.4. Live Tissue Imaging of GFP-E. coli Using IVIS Spectrum CT Biophotonic Imager in Ex Vivo Rat Skin

Rats were euthanized at 1, 3 and 24 h after wounding and treatments. The skin tissues surrounding the wound (~2 cm diameter and 1.5 cm deep) were sectioned and sliced through the center of the wound to visualize and quantify the width and depth of GFP fluorescence in the infected skin tissue that represent the spread of GFP-*E. coli* into the wound and surrounding tissues using the IVIS Spectrum CT Biophotonic Imager (Perkin Elmer, Waltham, MA, USA). GFP fluorescence was detected with an excitation wavelength of 500 nm, emission wavelength of 540 nm and a 2 s exposure time. All images were acquired at 73 μm spatial resolution. Intensity of GFP fluorescence in each wound and the depth and width of GFP fluorescence in each wound was quantified and analyzed in ImageJ (NIH, Bethesda, MD, USA).

### 2.5. Ex Vivo Porcine Skin Wounding, Infection and Determination of the Effect of DMTD Delivery on GFP-E. coli Penetration into the Wound Tissue

Pig skin tissue anatomy is very similar to that of human skin and it is used widely as a model for human skin [23,24,25]. In total, 10 cm × 10 cm full thickness skin samples were harvested from healthy, 6–9-month-old Yorkshire pigs (*Sus scrofa*) from the dorsal thoracic and dorsal lumbar areas. Harvested skin samples were further prepped by removing the hair on the surface and cutting into 1 cm^2^ pieces and these pieces were placed in Petri dishes. A shallow wound (0.5 mm deep with 9 mm^2^ area) was created using a scalpel. A 20 µL of GFP-*E. coli* culture (10^7^ cells) was placed into the wound and cells were allowed to attach for 45 min. A subset of the wounds were treated with 600 µL of saline containing 10 kDa Dextran Alexaflour 647 (DEX-AF-647 from ThermoFisher Scientific., D29914, 0.2 mg/mL). To visualize GFP-*E. coli*, the pig skin tissues were sliced through the center of the wound and the GFP fluorescence of the GFP-*E. coli* was imaged as in Section 2.4. Fluorescence from 10 kDa Dextran Alexaflour 647 (DEX-AF-647) was detected with an excitation wavelength of 675 nm, emission wavelength of 720 nm and a 10 s exposure time. All images were analyzed using ImageJ.

### 2.6. Quantification of GFP-E. coli Colony Forming Units (CFU) in the Infected Wounds of Rat

**Figure 4:**
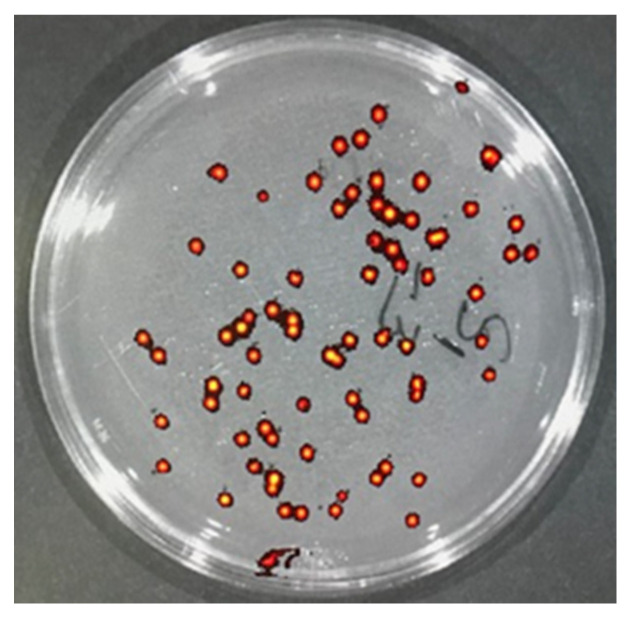
Representative image of LB agar-Amp plate on which a serial dilution of the bacteria from the skin tissue extract of a saline-treated wound was plated. All colonies exhibited robust GFP fluorescence, false colored yellow and red, when visualized using IVIS Spectrum CT Biophotonic Imager (excitation wavelength of 500 nm, emission wavelength of 540 nm, 2 s exposure time).

After visualization of the spread of GFP-*E. coli* in the infected skin tissue by IVIS Spectrum CT Biophotonic Imager, tissues were minced and placed into sterile tubes with 5 mL of sterile saline to extract bacteria. Briefly tubes with minced tissue and saline were vortexed briefly and rocked gently for 30 min at room temperature. Then, serial dilutions of the extracts were generated by diluting 20 µL of the extract into 1 mL of sterile saline in duplicates. These serial dilutions were plated on LB agar plates supplemented with ampicillin (100 µg/mL). After overnight incubation, colonies formed were authenticated by determining the presence of GFP fluorescence (Figure 4) and counted to determine the total CFU/mL of the extract by multiplying with dilution factor.

### 2.7. Determination of Vancomycin Tissue Distribution by LC-MRM

All mouse procedures were reviewed and approved by University of Missouri Institutional Biosafety Committee (IBC) and Animal Care and Use Committee (ACUC). Eight-week-old CD-1^®^ IGS female mice (The Charles River Laboratories, Wilmington, MA, USA) were procured and housed in ventilated cages and maintained on standard chow and water ad libitum at the University of Missouri animal facility.

Each mouse was anesthetized by isoflurane, its back was shaved and prepped by wiping with alcohol and a five mm-deep surgical incision was cut on top of the neck and sutured with 4-D nylon sutures. Mice were treated with 400 mg/kg vancomycin by either IP injection (*n* = 3) or DMTD delivery (*n* = 3) on top of the wounds. Two hours post-delivery, mice were euthanized, blood was collected and plasma samples were prepared. The skin around the DMTD delivery area was carefully wiped and then harvested along with kidney tissues. Harvested tissues were weighed and snap frozen in liquid nitrogen. Plasma, skin and kidney samples were subjected to liquid chromatography–mass spectrometric multiple reaction monitoring (LC-MRM) to quantify the concentration of vancomycin in these samples. An MRM transition consists of a precursor-fragment pair. The MRM approach allows the mass spectrometer (Thermo Scientific TSQ Quantiva QQQ) to ignore every molecule in a sample, except the compound of interest which facilitates accurate quantitation with very high specificity and sensitivity. To specifically quantify vancomycin a stock solution in water was infused and optimized conditions (ionization voltage, dwell time, collision energy) determined for 3 transitions using the compound optimization function in Quantiva Tune V2.0.1292.15 (Thermo Scientific, Waltham, MA, USA). Retention time was then confirmed on the C18 column by injecting 25 ng/mL of vancomycin.

To determine the vancomycin concentration in the plasma samples, the extraction was performed using a method modified from Oyaert et al., 2015 [26]. Briefly, 40 μL plasma was immediately vortexed with 40 μL IS working solution (1.8 ng/mL tolbutamide in 5% ACN/1% FA) and 160 μL acetonitrile added, to precipitate proteins, in Eppendorf tube. Samples were then centrifuged at 16K× *g*. In the IP-delivery group, replicate 1 diluted 1:5000 with internal standard solution (0.3 ng/mL tolbutamide in 5% ACN/1% FA) and replicate 2 and 3 diluted 1:1000 with internal standard solution. In the DMTD-delivery group, replicate 1, 2 and 3 diluted 1:10 with internal standard solution (0.3 ng/mL tolbutamide in 5% ACN/1% FA). Then, 100 µL was transferred into autosampler vials and placed in a cooled (7 °C) autosampler. To determine the vancomycin concentration in the skin and kidney tissues, the tissues were homogenized in 5-fold volumes of HPLC water. In total, 40 μL supernatant was vortexed with 40 μL IS working solution and 160 μL acetonitrile in an Eppendorf tube and centrifuged at 16K× *g*. In the skin IP-delivery group, replicate 1 and 2 were diluted 1:100 with IS solution and replicate 3 diluted 1:5000 with IS solution. In the skin DMTD-delivery group, replicate 1 and 2 diluted 1:1000 with IS solution and replicate 3 diluted 1:5000 with IS solution. In the kidney IP-delivery group, replicate 1 diluted 1:50,000 with IS solution, replicate 2 diluted 1:5000 with IS solution and replicate 3 diluted 1:10,000 with IS solution. In the kidney DMTD-delivery group, replicate 1 and 2 diluted 1:10 with IS solution, and replicate 3 diluted 1:100 with IS solution. Then, 100 µL was transferred into autosampler vials and placed in a cooled (7 °C) autosampler. Optimized LC-MRM was used to quantify vancomycin using the following parameters: A full-loop injection (10 µL) was loaded onto a C18 trap column (pepmap100, Eksigent, Redwood City, CA, USA). Compounds were eluted from the trap column and separated on a 20 cm × 75 µm inner-diameter pulled-needle analytical column packed with HxSIL C18 reversed phase resin (Hamilton Co., Reno, NV, USA) with a step gradient of acetonitrile at 500 nL/min. The Eksigent Nano 1D plus HPLC system is attached to a Thermo Scientific TSQ Quantiva triple-quadrupole mass spectrometer. LC gradient conditions: initial conditions were 2% B (A: 0.1% formic acid in water, B: 99.9% acetonitrile, 0.1% formic acid) for 2 min, followed by 8 min ramp to 90% B, hold at 90% B for 3 min, ramp back to (1 min) and hold at (4 min) initial conditions. Total run time was 18 min. MRM conditions: ionization voltage 1600 V, Q1 and Q3 resolution 0.7 (full-width half max), collision gas 1.5 mTorr and Dwell Time 200 msec. Data (mean and SD) for triplicate injections were then quantified (area under curve, sum of three transitions) using Xcalibur Quan Browser (V3.0.49, Thermo Scientific, Waltham, MA, USA) by comparison to standard curves of vancomycin in IS solvent.

### 2.8. Statistical Analysis

All experiments were performed at least in triplicates for each biological sample (GFP fluorescence of rat or pig skin tissue, GFP-*E. coli* CFU, vancomycin tissue concentrations) and at least in duplicates for each biological sample (tissue-extract dilutions to determine GFP-*E. coli* CFU). Statistical differences were estimated via either ANOVA or Student’s *t*-test.

## 3. Results

### 3.1. DMTD Delivery of CMS on Top of the Skin Effectively Attenuated Deep Skin Infection by GFP-E. coli in a Rat Model as Determined by GFP Fluorescence

In the clinic, only systemic delivery of CMS is used to treat deep skin infections with difficult-to-treat Gram-negative bacteria. CMS is a prodrug that is hydrolyzed to colistin, and colistin is the active molecule with bactericidal activity against Gram-negative bacteria [27]. Since CMS is 1251.5 daltons in size and molecules larger than 500 daltons cannot pass through skin, systemic delivery is the only way to get this drug to deep skin infections in closed wounds. However, colistin is toxic to vital organs and therefore an alternative method for localized skin delivery that can carry CMS to deep regions of skin is a critical unmet need. Therefore, we tested if the DMTD-delivered micromist containing CMS onto the skin above the deep skin infection would carry CMS to the site of infection (5 mm or more deep into the skin) and attenuate a Gram-negative bacteria infection.

**Figure 5:**
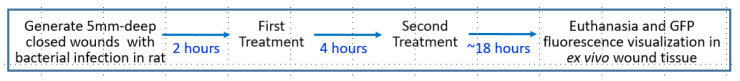
Timeline of wounding, treatments and GFP fluorescence visualization.

A closed wound model for deep bacterial infection was used here to test if the growth of a Gram-negative bacteria (GFP-*E. coli*) located at 5 mm or more deep into the skin can be attenuated by the micromist generated by DMTD that encapsulates CMS dissolved in sterile saline. The timeline of wounding and treatments is shown in Figure 5.

Each rat had three wounds on their back generated by the method shown in Figure 2: in total, 2 wounds on the left side and one wound on the right side. The average distance between the wounds was 5.43 ± 0.394 cm. The nozzle of DMTD had an outer diameter of 2 cm and inner diameter of 1.8 cm. This removable nozzle touched the skin surrounding the wound to fully cover the wound surface and prevent any escape of DMTD-delivered saline or CMS to other wounds. Two DMTD devices were used for micromist generation using DMTD for saline (negative control) and CMS solution to further ensure that there would be no cross-contamination.

**Figure 6:**
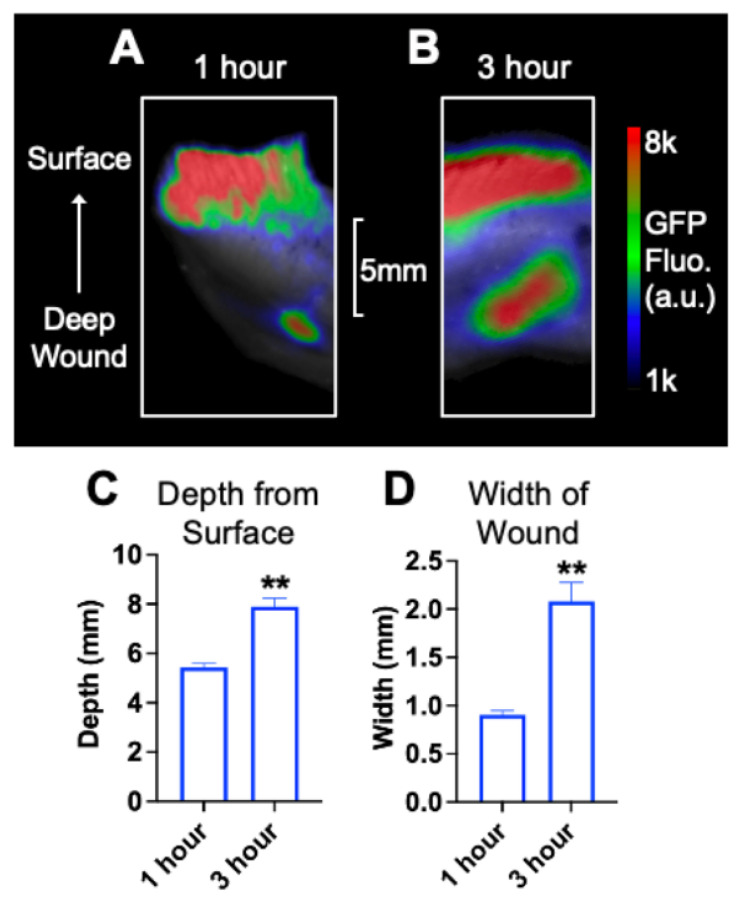
Representative images of deep skin wound tissue after 1 (**A**) and 3 (**B**) hours of initiating infection with GFP-*E. coli*. Histograms show the quantification of the depth (**C**) and width (**D**) of infection in the skin. *n* = 3 per group. a.u.: arbitrary units. ** *p* < 0.01.

Figure 6A,B show representative images the GFP fluorescence (509 nm) generated by GFP-*E. coli* in deep skin wounds of female Wistar rats after 1 h and 3 h of initiating infection. The infection was initiated by placing 2.3 × 10^9^ GFP-*E. coli* cells. The average depth (Figure 6C) and width (Figure 6D) of GFP fluorescence was 5.4 ± 0.3 mm and 0.9 ± 0.1 mm, respectively, after one hour.

This indicates that GFP-*E. coli* infection was present at a depth of >5 mm from the top of the skin. The average depth and width of GFP fluorescence was 7.9 ± 0.7 mm and 2.1 ± 0.4 mm, respectively, after three hours of inducing the infection (Figure 6C,D). This observation further confirms the presence of robust *E. coli* infection after 3 h of introducing these bacteria to the deep skin wound.

**Figure 7:**
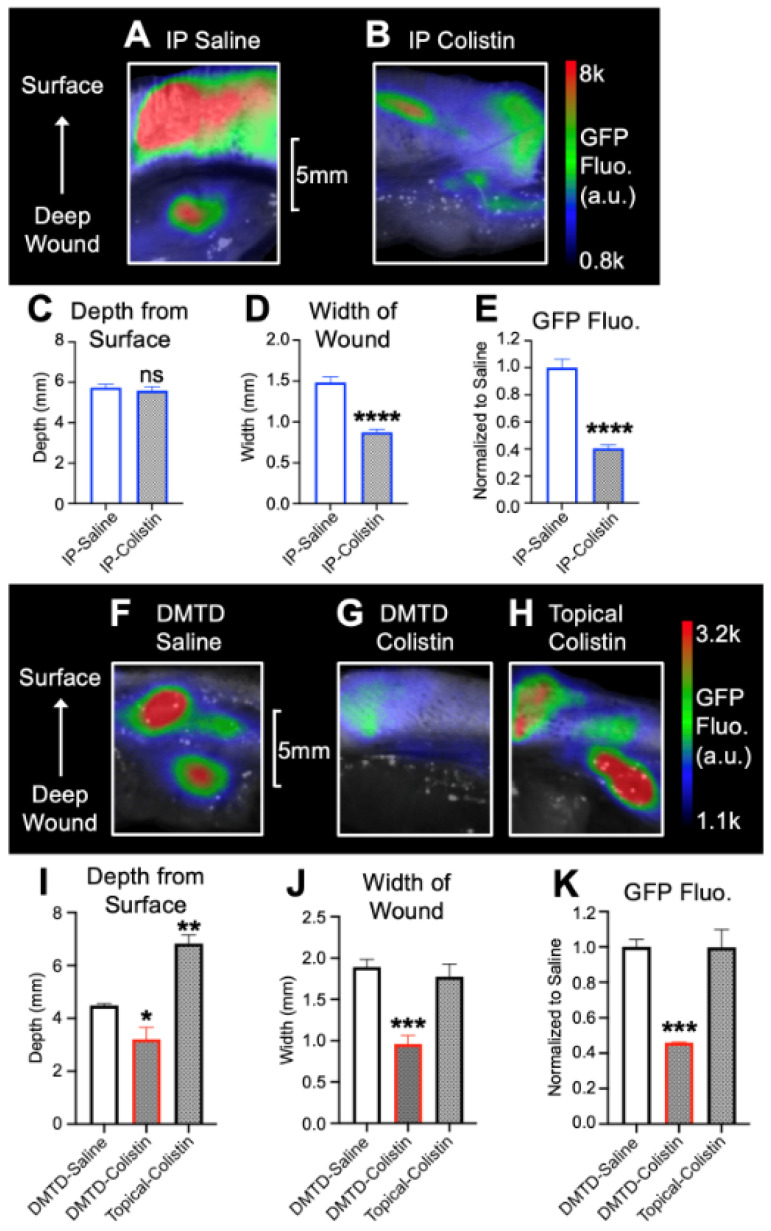
Representative images of rat wound tissue infected with GFP-*E. coli* and treated with intraperitoneal saline (IP-saline: (**A**)) or CMS (IP-colistin: (**B**)). The depth of the wound from the top of the skin (surface) is marked. Histograms (**C**,**D**) show the changes in the depth and width of infection after IP-colistin treatment and (**E**) shows the overall intensity of the GFP fluorescence in IP-colistin wounds compared to IP-saline wounds (normalized to IP-saline wounds). (**F**,**G**) show representative images of rat wound tissue infected with GFP-*E. coli* and treated with the micromist delivered by DMTD that carries either saline ((**F**): DMTD saline), or CMS dissolved in saline ((**G**) DMTD colistin) to the deep skin tissue. (**H**) (Topical colistin) shows representative image of wound tissue infected with GFP-*E. coli* and treated with CMS (same dose) delivered on top of the skin by a micropipette and retained on the skin (above the infected wound) by a barrier created by ophthalmic ointment. Histograms (**I**,**J**) show the changes in the depth and width of infection after treatments with DMTD-delivered micromist carrying saline or CMS. Histogram (**K**) shows the overall intensity of the GFP fluorescence in wounds treated with DMTD-colistin compared to DMTD-saline or topical colistin treatments (normalized to DMTD-saline wounds). *n* = 6 for each treatment group in (**A**–**E**). *n* = 5 for each treatment group in F-K. fluo.: fluorescence a.u: arbitrary units. * *p* < 0.05; ** *p* < 0.01; *** *p* < 0.001, **** *p* < 0.0001, ns: not significant *p* > 0.05.

We used intraperitoneal (IP) injection of CMS as the mode of systemic delivery of CMS to rats with deep skin infections. Figure 7A,B show representative images of wound tissues from rats treated with IP delivery of either saline or CMS (2.5 mg/kg in 300 µL). The depth of robust GFP fluorescence present in the wound tissue in the rats treated with IP delivery of saline had an average of 5.7 ± 0.4 mm from the top of the skin after 24 h (Figure 7C). Treatment with IP-CMS (IP-Colistin in Figure 7B,C,E) did not significantly change the depth of GFP fluorescence (Figure 7C), However, the width of GFP fluorescence was significantly suppressed by treatment (Figure 7D). Moreover, there was an overall significant 2.5-fold reduction in the GFP fluorescence in their wound tissues (Figure 7B,E). Thus, IP-delivered CMS at a dose of 2.5 mg/kg could attenuate Gram-negative bacterial (GFP-*E. coli*) infection located at a depth of >5 mm from the top of the skin in our rat model for deep wound with infection after 24 h. This served as the positive control for the dose and duration of systemic CMS treatment that can successfully attenuate deep skin Gram-negative bacterial infection in our rat model for deep skin infection. The same dose and duration of CMS treatment was used for the studies to test if DMTD-delivered micromist containing CMS could also effectively attenuate deep skin Gram-negative bacterial infection shown in Figure 7F–K.

Figure 7F shows the representative image of GFP fluorescence in a rat wound treated with saline micromist delivered via DMTD (vehicle control). This serves as the control for untreated deep skin infection. Figure 7G shows the representative image of GFP fluorescence in a rat wound treated with the micromist delivered by DMTD that carries CMS (2.5 mg/kg in 300 µL, delivered in a 30 s time period). Figure 7H shows the representative image of GFP fluorescence in a rat wound treated with an equal amount of CMS (2.5 mg/kg) in 100 µL of saline placed on top of the wound (referred as topical colistin hereafter). The 100 µL CMS solution was placed on top of the skin surrounding the infection site using a micropipette and retained by a barrier created with ophthalmic ointment (ndc24208-780-55) to prevent loss of CMS. This topical colistin treatment serves as the negative control since CMS has a high molecular weight and cannot go through skin when delivered on top of the skin located above a deep skin infection. We used ndc24208-780-55 for creating the barrier to prevent CMS loss since it is approved for use on rats in our IACUC protocol (B2020-56, approved 23/06/2020). The ingredients that could have contributed to the occlusive properties of this ointment are mineral oil and petroleum.

There was significant reduction in the average depth (Figure 7I) and width (Figure 7J) of GFP fluorescence in the DMTD-colistin wounds compared to DMTD-saline or topical saline wounds. These data indicate that DMTD-delivered micromist containing CMS locally on top of the skin could effectively attenuate GFP-*E. coli* infection in the deep tissue located at 5–6 mm from the top of the skin. The reduction in the overall intensity of GFP fluorescence by DMTD-CMS treatment (Figure 7K) was similar to that of IP-CMS treatment (Figure 7E).

### 3.2. DMTD-Delivery of CMS on Top of the Skin Effectively Attenuated Deep Skin Infection by GFP-E. coli in a Rat Model as Determined by Colony Fromining Unit (CFU) Quantification

**Figure 8:**
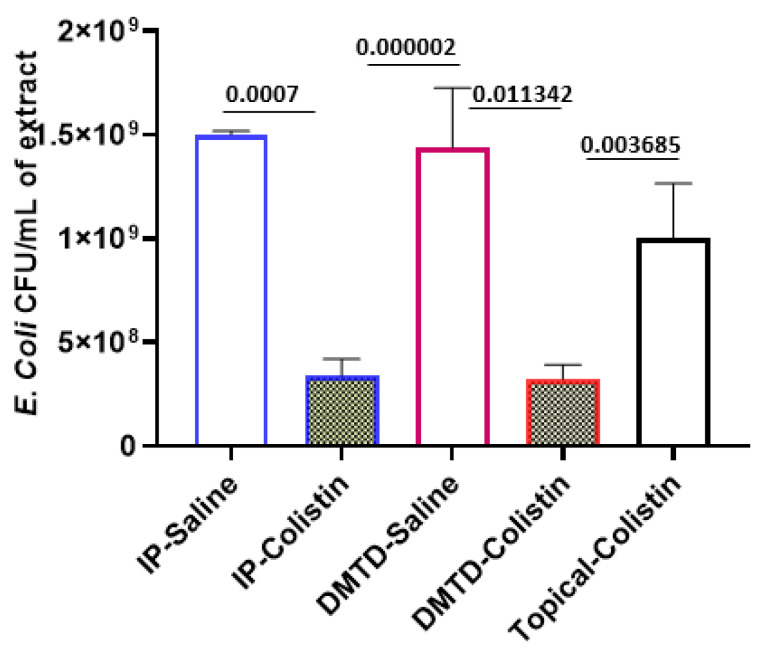
Total number of CFUs present in the 5 mL of tissue extracts of infected deep skin wounds that were treated with IP-delivered saline (IP-saline) or colistin (IP-colistin), DMTD-delivered saline (DMTD-saline) or colistin (DMTD-colistin) and topical application of equal dose of colistin (Topical colistin). The actual *p* values are shown on top of the bars.

Figure 8 shows the total CFU of GFP-*E. coli* present in the infected skin wound tissue extracts from all treatment groups. The average number of CFUs recovered from wounds treated with IP-delivered (*n* = 9) and DMTD-delivered (*n* = 4) saline was in the range of 1.5–2 × 10^9^ cells.

The average number of GFP-*E. coli* CFUs recovered from wounds treated with IP-delivered (*n* = 9) and DMTD-delivered (*n* = 6) CMS was 3.4 ± 0.8 × 10^8^ and 3.23 ± 0.6 × 10^8^, respectively, indicating that systemic IP-delivered and localized DMTD-delivered CMS were similarly effective in attenuating the robust GFP-*E. coli* infection in the infected wounds in the female Wistar rat model. The average number of GFP-*E. coli* CFUs recovered from wounds (*n* = 4) treated by topical application with an equal dose of colistin (2.5 mg/kg) as that used in IP or DMTD delivery, but in a smaller volume (100 µL) placed in a barrier made of ophthalmic ointment, was 1 ± 0.2 × 10^9^ and this was not statistically significantly different from that of saline-treated wounds.

### 3.3. DMTD Delivery Approach Does Not Cause Deeper Penetration of Bacteria in Infected Wounds on Ex Vivo Yorkshire Pig Skin

**Figure 9:**
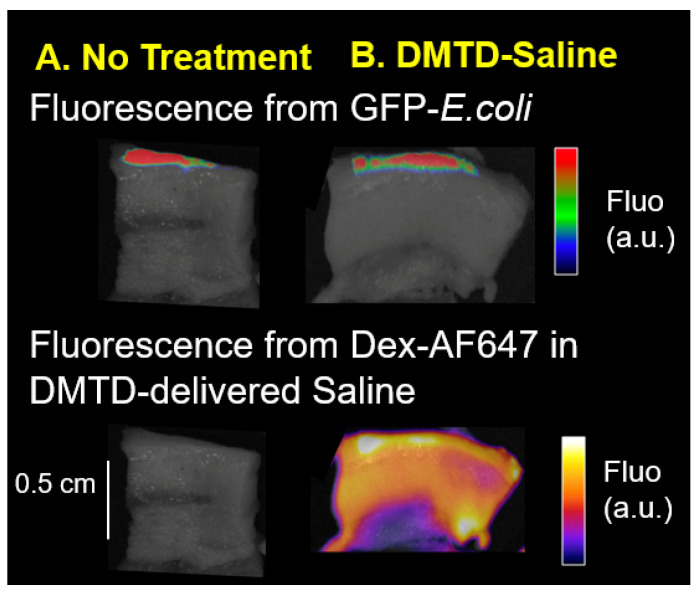
Representative images of ex vivo pig skin wounds infected with GFP-*E. coli*. The depth of GFP fluorescence was similar in wounds receiving 1 min delivery of 600 µL of DMTD-delivered saline ((**B**): *n* = 3) and the undisturbed wound ((**A**): no treatment: *n* = 3). DEX-AF-647 in the DMTD-delivered saline reached 5 mm depth in the skin indicating that the DMTD delivery was successful. a.u. Arbitrary Units; Fluo: Fluorescence.

Since DMTD-delivered micromist can reach several millimeters deep into the skin, an important concern is if DMTD-delivered micromist will cause deeper penetration of microbes present on the wound surface causing deeper penetration of infection. Therefore, we determined if DMTD-delivered saline caused deeper penetration of bacteria present on the surface of a wound using ex vivo Yorkshire pig skin that is anatomically similar to human skin [23,24,25] and GFP-*E. coli* to easily track the depth of penetration of the bacteria. We generated open shallow wounds (0.5 mm deep 9 mm^2^ surface area) on ex vivo pig skin and infected them with 2 × 10^7^ GFP-*E. coli* cells as described in Section 2. After 45 min (allowing bacteria to attach), images of wounds were taken to visualize GFP fluorescence using IVIS Spectrum CT Biophotonic Imager, Perkin Elmer, Waltham, MA, USA. GFP fluorescence was visible at the wound surface (Figure 9A: No Treatment). Then, a subset of wounds (*n* = 3) were subjected to a 1 min DMTD delivery of 600 µL of saline containing DEX-AF-647 to track the depth of delivery. However, DMTD delivery of saline did not increase the depth of GFP fluorescence (from GFP-*E. coli*) indicating that 1 min DMTD delivery of saline micromist did not result in deeper penetration of GFP-*E. coli* into the skin. The fluorescence from DEX-AF-647 present in the DMTD-delivered saline reached a depth of ~5 mm into the pig skin (Figure 9B) indicating that the micromist delivered by DMTD reached deep skin tissue as expected.

### 3.4. DMTD Delivery Approach Does Not Cause Deeper Penetration of Bacteria in Infected Full Thickness Open Wounds on Female Wistar Rats

**Figure 10:**
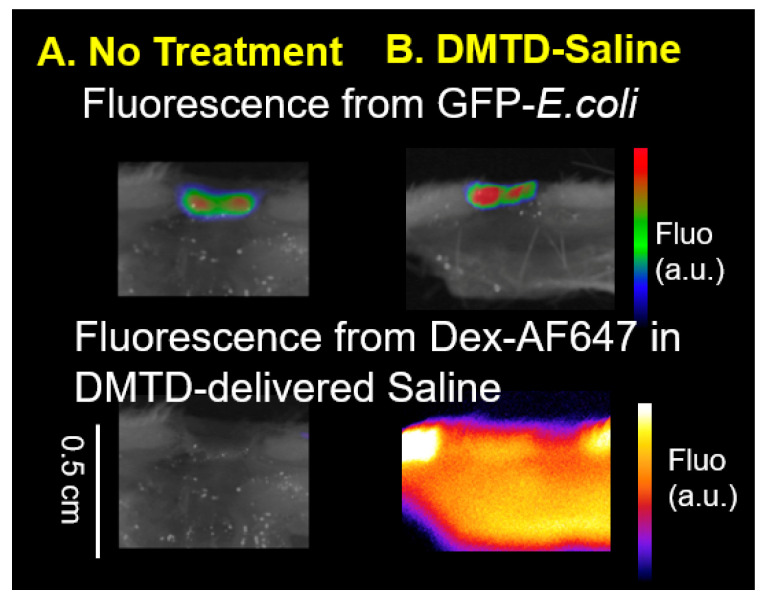
Representative images of rat skin wounds infected with GFP-*E. coli.* The depth of GFP fluorescence was similar in untreated wounds (**A**) and wounds treated with 1 min delivery of 600 µL of DMTD-delivered saline containing the fluorochrome DEX-AF-647 (**B**). *n* = 3 for all groups. DEX-AF-647 in the DMTD-delivered saline reached 5 mm depth in the skin indicating that the DMTD delivery was successful. a.u. Arbitrary Units; Fluo: Fluorescence.

To further confirm that DMTD-delivered micromist does not cause deeper penetration of bacteria in the wounds of a live animal model, we used a rat model with infected open wounds. We infected 4 mm full thickness open wounds located on the back of female Wistar rats with GFP-*E. coli*. Infection with GFP-*E. coli* was carried out by placing 20 µL of GFP-*E. coli* cells (2 × 10^7^ cells) on the open wound with a micropipette 48 h after the wound was made. This time lapse was to ensure that the wounds were not bleeding anymore and were partially healed, and that the bacteria could attach well to the wound surface without getting washed away by the wound exudate. After 45 min, a subset of wounds were subjected to 1 min DMTD delivery of 600 µL of saline containing DEX-AF-647. Then, the rats were euthanized and the skin tissue was dissected out and imaged using IVIS Spectrum CT Biophotonic Imager. As shown in Figure 10A, GFP fluorescence was detected on the undisturbed wound surface of rat skin and the depth of GFP fluorescence was similar in the wound that received 1 min DMTD delivery of 600 µL of saline. However, as in the pig skin, the micromist delivered by DMTD reached up to a depth of ~5 mm in the skin as evidenced by the Dex-AF-647 fluorescence. Thus, DMTD-delivered micromist could reach deep into the skin without causing the spread the bacterial infection.

### 3.5. DMTD-Delivered Vancomycin on Top of the Skin Reached Similar Skin Concentrations as IP-Delivered Vancomycin, but Did Not Cause Accumulation of the Drug in the Plasma and Kidney

Vancomycin is the initial antibiotic of choice against hard-to-treat Gram-positive bacteria [28,29]. However, systemic application of vancomycin causes vital organ damage, particularly nephrotoxicity [30,31]. To determine if DMTD-delivered vancomycin on top of the skin results in the entry to the bloodstream similar to a systemic delivery and accumulates in the kidney that can cause nephrotoxicity, we compared tissue partitioning of DMTD-delivered and IP-delivered vancomycin in a mouse model.

**Figure 11:**
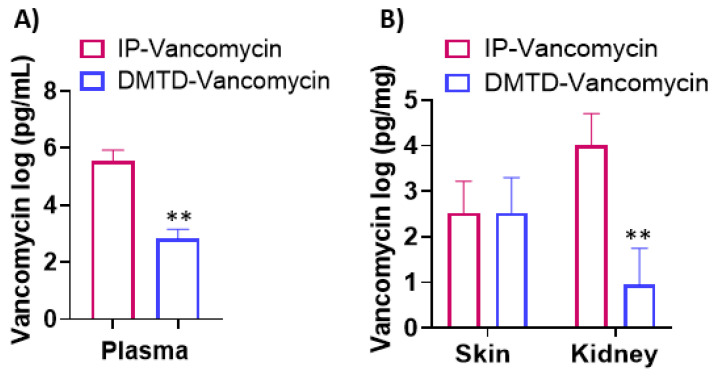
Vancomycin levels in (**A**) Plasma and (**B**) skin and kidney when the drug was delivered by either systemic IP or localized DMTD delivery to mice. *n* = 3. ** *p* < 0.01.

The vancomycin dose used was 400 mg/kg that corresponds to the median lethal intravenous dose for mice [29]. Two hours after delivering the vancomycin via DMTD (*n* = 3) or IP (*n* = 3), the mice were euthanized and the vancomycin concentration in the plasma, skin and kidney was measured using LC-MRM technique. During the 2 h washout period, the mice were active and eating. As shown in Figure 11, the vancomycin levels in skin tissues treated with IP or DMTD delivery of the antibiotic were comparable, suggesting that the DMTD could successfully deliver vancomycin to skin with equivalent efficiency as systemic delivery. Conversely, the vancomycin levels in the mice treated with DMTD delivery on top of the skin had about 1000-fold lower concentration of vancomycin in the plasma and kidney compared to the mice that received IP delivery of vancomycin. This observation indicates that DMTD-delivered vancomycin locally on top of the skin does not result in high concentrations of vancomycin in the plasma and kidney that can lead to severe vital organ damage and lethality.

## 4. Discussion

Wound and burn infections by multidrug-resistant (MDR) bacteria are a major and growing disease burden. Due to the high risk of septicemia and death in patients, early treatment is essential. The increase in antibiotic resistance globally necessitates the use of reserve antibiotics such as colistin and vancomycin. Colistin in particular has re-emerged as an effective antibiotic for Gram-negative bacterial infections [19,32,33]. Colistin methanesufonate (CMS), the prodrug of colistin, is preferred over colistin in clinic because CMS is less toxic than colistin when administered parenterally. In clinic, CMS and vancomycin are delivered only by systemic delivery to attenuate hard-to-treat deep skin and soft tissue infections because these high molecular weight drugs cannot penetrate stratum corneum and/or migrate through several millimeters of skin to reach the site of deep skin infection. Thus, while systemic administration of these broad-spectrum antibiotics (CMS and vancomycin) is the last line of defense against deep wound infections by MDR bacteria, the high dosage required, limited tissue partitioning and toxicity concerns highlight the need for methods to increase their effectiveness. Up to 21% of patients hospitalized for acute skin infections have mixed Gram-positive and Gram-negative pathogens in the wound bed [34]. While colistin and vancomycin co-administration via intravenous route has been demonstrated to effectively treat these mixed culture infections, the high risk of toxicity has limited this treatment method to the critically ill patients [34]. Both drugs have been demonstrated to effectively treat surface-level skin and eye infections without any impact on healing times or notable toxicity [35].

The inability of CMS and vancomycin (both over 1000 daltons) to penetrate into the skin and tissue renders topical formulations of these drugs ineffective in treating deep wound and tissue infections. Droplette micromist technology generates an enhanced, high-velocity aerosol in a transitional flow that is composed of many sub-micron droplets delivered via low-pressure acceleration towards areas of the skin surface (not a single needlepoint) to achieve deep tissue delivery of particles up to five megadaltons in molecular weight. The technology is molecule-agnostic and is rather limited by fluid viscosity and surface tension. Both CMS and vancomycin are water soluble and readily compatible with the custom built Droplette micromist technology device (DMTD).

To compare tissue partitioning of high molecular weight antibiotics delivered via DMTD or IP, we determined the levels of vancomycin delivered by DMTD or IP in the skin, kidney and serum of mice after a 2 h washout period. Vancomycin at a dose of 200 mg/kg is known to cause changes in gene expression that leads to nephrotoxicity [30] and at a dose of 400 mg/kg is known to cause severe nephrotoxicity [31]. Therefore, we used the higher dose of vancomycin (400 mg/kg) to determine how much of the locally delivered vancomycin by DMTD accumulates in the blood and kidney, compared to that of systemic delivery of vancomycin. There were significantly higher levels of vancomycin in kidney and plasma by the systemic IP delivery compared to DMTD delivery (*p* < 0.01), indicating much less likelihood of tissue damages caused by localized DMTD delivery of vancomycin. The skin vancomycin levels, on the other hand, were comparable between the two methods. Therefore, localized DMTD delivery of vancomycin is a safer approach that can result in similar vancomycin skin concentration as that of systemic delivery without causing increased plasma concentrations of vancomycin and vital organ toxicity. Moreover, localized DMTD delivery of vancomycin will enable bypassing first-pass metabolism and is expected to result in lower nephrotoxicity.

We recognize that the quantification of vancomycin in blood, kidney and skin after two hours is only partially informative about the drug distribution after local drug delivery by DMTD and this is a limitation of this study. Therefore, our future studies will include pharmacokinetics studies to compare the amount of both vancomycin and CMS in plasma and different tissues at different time points in different animal models in response to systemic and DMTD delivery of these drugs. That will help to obtain a more comprehensive picture of the safety of these drug delivery approaches. Nevertheless, our 2 h data on vancomycin tissue partitioning after DMTD and IP delivery highlight the fact that DMTD delivery effectively prevents the accumulation of toxic antibiotics used for treating skin infections in the blood and vital organs by about 1000-fold compared to systemic delivery.

In this study, we used a rat model for deep skin infection with GFP-*E. coli* to visualize the depth of infection with high sensitivity by quantifying the GFP fluorescence in the wounds. This model allowed us to determine the depth and width of infection at different time points and in response to IP-delivered and DMTD-delivered saline and CMS. This model exhibited robust GFP-*E. coli* infection at a depth of 6~7 mm that could be easily visualized by the presence of strong GFP signal after 1 and 3 h of initiation of infection (Figure 6). Both IP-delivered and DMTD-delivered CMS strongly suppressed the infection in these wounds (Figure 7), but interestingly, DMTD-delivered CMS completely suppressed infection within a 3–5 mm depth: GFP signal was visible only up to a 3 mm depth after treatment with DMTD-delivered CMS (Figure 7G,I). Conversely, although IP-delivered CMS is expected to reach deep skin tissue and kill bacteria effectively, we found that there was some GFP signal remaining at the depth of 5–6 mm from the top of the wound surface in the wounds treated with IP-delivered CMS (Figure 7B,D). However, the overall intensity of the GFP signal reduced similarly in the wounds treated with either IP- or DMTD-delivered CMS (Figure 7E,K). Moreover, the GFP-*E. coli* CFUs recovered from the wounds treated with either IP- or DMTD-delivered CMS were comparable (Figure 8). Thus, the overall suppression of infection by systemically delivered and DMTD-delivered CMS was comparable. However, the difference in the ability of IP-delivered and DMTD-delivered CMS in killing *E. coli* located in the deep part of skin tissue may suggest that DMTD-delivered CMS reaches these locations more effectively than IP-delivered CMS. This could simply be due to some microcirculation-related impairment that may have reduced the access of systemically delivered CMS present in the blood to reach all sites where bacteria were located in the deep skin tissue. Additional studies are warranted to further explore if DMTD-delivered CMS reaches deep skin tissue more effectively and kill bacteria better than IP-delivered CMS.

Data presented here show that DMTD delivery of CMS, a bactericidal drug for Gram-negative bacteria, on top of the skin around a closed wound with deep skin infection with GFP-*E. coli* can effectively attenuate infection. We have used GFP-*E. coli* here as a model for Gram-negative bacteria that can be tracked easily due to the GFP signal although this *E. coli* strain is not MDR. Nevertheless, since systemic administration of CMS is the last line of defense against MDR Gram-negative bacteria, our data show that DMTD-delivered CMS attenuates an *E. coli* infection located >6 mm deep in the skin (Figure 7D,I), suggesting that DMTD-delivered CMS can be effective in treating deep skin infections by MDR Gram-negative bacteria. Further studies are warranted to confirm this idea.

While it was promising to see DMTD delivery of CMS attenuated deep skin infections, one recurring concern was whether the mechanical means of delivery can also push bacteria deeper into the wound bed. To test this, we created open wounds on ex vivo pig skin that mimics human skin, and on live rat skin, and infected these wound beds with GFP-*E. coli*. Since GFP signal is a highly sensitive marker for the presence of GFP-*E. coli*, we then measured the depth of penetration of GFP-*E. coli* after the wound and surrounding skin were sprayed with DMTD-delivered saline for 1 min. We did not observe any changes in the depth of GFP signal indicating that the micromist delivered by DMTD does not encapsulate bacteria and carry it further deep into the skin. Since the saline micromist contained a fluorochrome, DEX-AF-647, we could track how deep into the skin this micromist could reach. As expected, the micromist reached ~5 mm deep into the skin. Therefore, we propose that DMTD delivery of high molecular weight antibiotics on top of the skin does not cause deeper penetration of bacteria and DMTD delivery is a safe method for localized, needle-free, treatment of deep skin infections.

DMTD-mediated delivery of these antibiotics offers several advantages over topical application. DMTD is designed to achieve deeper penetration into skin and tissue, thus allowing the antibiotic to reach deeper into the wound bed. Since DMTD delivery is needle free and the micromist does not cause pain, this is a preferable method to injections to treat deep skin infections. Additionally, the device dispenses pre-metered volumes that are packaged into single-use capsules to enable precise dosing. These capsules are RFID (Radio Frequency Identification) tagged, enabling the device to modify its spray settings in real-time to optimize for the volume and intended depth of delivery. While future studies will be performed to fully quantify the variance in delivery amount and depth, in these experiments two variables were controlled to create as consistent delivery as possible. First, the use of cartridges containing pre-metered volumes of the active molecules mitigates against the risk of under-delivery or over-delivery. Second, the use of a nozzle that makes direct contact with skin and covers the entire wound and surrounding skin mitigates against spray-off and ensures that all of the drug reaches the skin. This technology also enables easy co-delivery of various antibiotics together or with analgesics or other wound-healing agents. Finally, delivery directly into tissue enables bypassing first-pass metabolism and reduces the toxicity risks of the antibiotics. While further studies are required, we hypothesize that in patients with impaired microcirculation and deep skin infection, DMTD delivery of broad-spectrum antibiotics that have high molecular weights and systemic toxicity effects may enable larger volumes of the antibiotic to reach the deep wound bed compared to parenteral delivery methods and without systemic toxic effects.

## Data Availability

The original contributions presented in the study are included in the article.
